# Biological Activities of Extracts from Loquat (*Eriobotrya japonica* Lindl.): A Review

**DOI:** 10.3390/ijms17121983

**Published:** 2016-12-06

**Authors:** Yilong Liu, Wenna Zhang, Changjie Xu, Xian Li

**Affiliations:** 1 Zhejiang Provincial Key Laboratory of Horticultural Plant Integrative Biology, Zhejiang University, Zijingang Campus, Hangzhou 310058, China; yilongliu@zju.edu.cn (Y.L.); nawen2007@163.com (W.Z.); chjxu@zju.edu.cn (C.X.); 2 Catch Bio-Science & Technology Co., Ltd., 5F of Building G, No. 1 North Guotai Road, Zhangjiagang 215600, China; 3 The State Agriculture Ministry Laboratory of Horticultural Plant Growth, Development and Quality Improvement, Zhejiang University, Zijingang Campus, Hangzhou 310058, China

**Keywords:** *Eriobotrya japonica* Lindl., bioactivities, bioactive compounds

## Abstract

Loquat (*Eriobotrya japonica* Lindl.) is a subtropical fruit tree with high medicinal value native to China. Different organs of loquat have been used historically as folk medicines and this has been recorded in Chinese history for thousands of years. Research shows that loquat extracts contain many antioxidants, and different extracts exhibit bioactivity capable of counteracting inflammation, diabetes, cancer, bacterial infection, aging, pain, allergy and other health issues. Bioactive compounds such as phenolics and terpenoids have been isolated and characterized to provide a better understanding of the chemical mechanisms underlying the biological activities of loquat extracts. As the identification of compounds progresses, studies investigating the in vivo metabolism, bioavailability, and structure–activity relationships, as well as potential toxicity of loquat extracts in animal or cell models are receiving more attention. In addition, genetic studies and breeding of loquat germplasms for high contents of health-benefiting compounds may provide new insight for the loquat industry and research. This review is focused on the main medicinal properties reported and the possible pharmaceutically active compounds identified in different loquat extracts.

## 1. Introduction

Loquat (*Eriobotrya japonica* Lindl.) is a subtropical evergreen fruit tree originating in southeastern China. It has been cultivated for more than 2000 years in China and is now commercially cultivated in more than 30 countries worldwide, including Japan, Turkey, Brazil, Spain, India, Pakistan, Israel, and Italy. China is now the largest producer of loquat fruit with a cultivation area of about 170,000 ha and an annual output of about one million tons. The fruit ripen from May through June in the main Chinese production areas such as Zhejiang, Fujian, and Jiangsu provinces, earlier than the majority of other domestically-grown fruits. Therefore, loquat provides an extended dietary source of fresh fruit for Chinese consumers and a high economic return to producers due to the lack of competition within this market niche. There are two main types of loquat fruit, i.e., red-colored and white-colored cultivars, where different carotenoids accumulations cause the fruit color and nutritional differences [1].

Loquat is a plant with high medicinal value and different organs that have been used historically as folk medicines for thousands of years. Loquat extracts have been used for the treatment of cough, chronic bronchitis (CB), inflammation, diabetes, and cancer in Chinese folk medicine. Ancient literature, such as the ‘Compendium of Materia Medica’ [2], described the origin, classification, breeding methods, and medicinal value of the loquat tree, and laid the foundation for the development and cultivation of loquat.

The efficacy of loquat, as used in traditional Chinese medicine, is supported by current scientific evidence regarding the pharmacologically active compounds in plant extracts and their structure–activity relationships. The phytonutritional composition of extracts of different organs varies considerably: loquat leaf and flower are rich in phenolics and triterpenes; fruit is rich in sugars, organic acids, carotenoids, flavonoids, phenolic acids, and vitamins; the kernel is a good source of proteins, starch, tannins, and minerals [3,4,5]. Different loquat extracts have been shown to exhibit a wide range of activities. To provide a comprehensive understanding of the current research on the health-promoting effect of loquat extracts, the reported biological activities as well as the key bioactive compounds are summarized in the present review.

## 2. Bioactivities of Loquat Extracts

### 2.1. Anti-Inflammatory Activity

In Chinese folk medicine, loquat leaf has been used since ancient times to treat inflammatory diseases such as cough, CB, and asthma [2]. Modern scientific studies using different experimental models have proved the anti-inflammatory capacity of different loquat tissues such as leaf [6,7,8,9,10], seed [11,12] and fruit [13].

**Figure 1:**
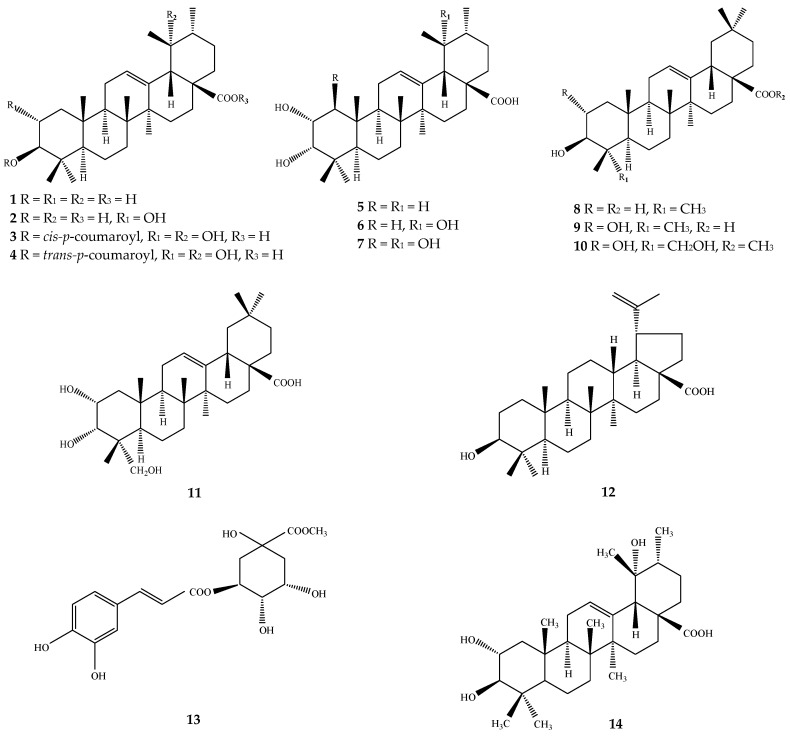
Structures of chemical compounds **1**–**14**. **1**: ursolic acid; **2**: corosolic acid; **3**: 3-*O*-*cis*-*p*-coumaroyltormentic acid; **4**: 3-*O*-*trans*-*p*-coumaroyltormentic acid; **5**: 3-epicorosolic acid; **6**: euscaphic acid; **7**: 1β-hydroxyeuscaphic acid; **8**: oleanolic acid; **9**: maslinic acid; **10**: methyl arjunolate; **11**: 2α,3α,23-trihydroxyolean-12-en-28-oic acid; **12**: betulinic acid; **13**: methyl chlorogenic acid; **14**: tormentic acid.

Pulmonary inflammation is a factor in many lung diseases. Lipopolysaccharide (LPS)-induced inflammation is a common experimental model for anti-inflammatory research. Loquat leaf extracts enriched with triterpene acids, especially ursolic acid (**1**), showed anti-inflammatory effects on alveolar macrophages in rats with LPS-induced CB [7,8,14,15]. Twelve triterpene acids, e.g., seven ursane-type [ursolic acid (**1**), corosolic acid (**2**), 3-*O*-*cis*-*p*-coumaroyltormentic acid (**3**), 3-*O*-*trans*-*p*-coumaroyltormentic acid (**4**), 3-epicorosolic acid (**5**), euscaphic acid (**6**), 1β-hydroxyeuscaphic acid (**7**)], four oleanane-type [oleanolic acid (**8**), maslinic acid (**9**), methyl arjunolate (**10**), 2α,3α,23-trihydroxyolean-12-en-28-oic acid (**11**)], and one lupane-type [betulinic acid (**12**)] isolated from the ethyl acetate-soluble fraction of loquat leaf showed marked anti-inflammatory effects in the inhibition of 12-*O*-tetradecanoylphorbol-13-acetate (TPA)-induced ear edema of mice, and the 50% inhibitory dose of these twelve compounds ranged from 0.03–0.43 mg per ear (Figure 1) [6]. The mouse paws edema model was also used to assess the anti-inflammatory effect of loquat extract [16,17], and loquat tea extract made from roasted fresh loquat leaves significantly decreased the paw edema of mouse [16].

Loquat seed extracts also showed anti-inflammatory effects in vivo [11,12]. By using a chemotherapy drug (5-fluorouracil)-induced mucositis model in hamsters, the loquat seed extract significantly inhibited the chemotherapy-induced mucositis, and the epithelial injury and bacterial infection were greatly inhibited, together with much lower plasma lipid peroxide level [11]. In another study, by using a dinitrofluorobenzene-induced allergic dermatitis in rat ear as an experimental model, administration of loquat seed extract resulted in significantly inhibited development of allergic dermatitis, where lower ear thickness and serum immunoglobulin E levels as well as improved balance of Th1/Th2 were observed [12].

In addition, loquat juice also showed anti-inflammatory effects. Fruit juice was administrated prophylacticly, postmortemly or concurrently with LPS stimulation, and was found to exhibit a prophylactic effect on LPS-induced inflammation in peritoneal macrophages [13].

Increased levels of inducible nitric synthase (iNOS), cyclooxygenase-2 (COX-2), and pro-inflammatory cytokines such as interleukin-1β (IL-1β), tumor necrosis factor-α (TNF-α) and interleukin-8 (IL-8) have been correlated with inflammation [8,14,18]. Therefore, decreasing pro-inflammatory mediators (such as iNOS, COX-2, TNF-α, IL-6, IL-8, IL-1β) and/or increasing anti-inflammatory cytokine (such as IL-10) secretions are important mechanisms for the anti-inflammation effects of loquat extracts [8,13,14,18,19,20,21]. Such regulation was associated with suppressing the expression and activation of the nuclear factor-κB (NF-κB) [18,19,22,23,24] and/or mitogen-activated protein kinase (MAPK) signaling pathway [15,22,23], which have been suggested as key regulators of the expression of inflammatory mediators in the cellular signaling pathway [25,26].

Treatments such as LPS induce production of iNOS, COX2, TNF-α, IL-1β, and IL-8 in A-549 human lung epithelial cells and loquat leaf extract and its triterpene ursolic acid (**1**) inhibited the LPS-induced cytokines and the inducible enzyme production via the NF-κB signaling pathway in A-549 cells [18]. The anti-inflammatory effect of loquat leaf extract might result from the inhibition of expression of iNOS and COX-2 through the downregulation of NF-κB activation and MAPK phosphorylation in LPS stimulated RAW264 cells [23] and in the LPS-activated murine peritoneal macrophage model [24]. Similarly, the anti-inflammatory effects of loquat tea extract may rely on the inhibition of the production of iNOS, nitric oxide (NO), IL-6, TNF-α, and on the downregulation of the transforming growth factor-β (TGF-β)-activated kinase-mediated MAPK and NF-κB pathways in mouse macrophage-like RAW 264.7 cells [16]. Mast cells induce the biosynthesis of pro-inflammatory cytokines with immune regulatory properties. Loquat leaf extract inhibited the secretion of TNF-α, IL-6, and IL-8 and attenuated the activation of NF-κB, p38 MAPK and extracellular signal-regulated kinase (ERK) in horbol 12-myristate 13-acetate and calcium ionophore A23187-induced mast cells [22]. More in-depth studies using microarray analysis showed that loquat leaf extracts inhibited the expression of a wide variety of inflammation-related genes in LPS-stimulated human gingival fibroblasts [9].

In animal models, loquat leaf extracts were also found to inhibit the NF-κB activation of alveolar macrophages, which led to the inhibition of the expression of TNF-α, IL-1β, prostaglandin E_2_ and leukotriene B_4_ in a dose dependent manner in CB rats [8,14]. In another study, triterpene acids extracts of loquat leaf were found to significantly inhibit the increase in NO concentration and iNOS expression, which may be related to the inhibition of phosphorylation of p38 MAPK and the corresponding signal transduction in alveolar macrophages of CB rats [15]. Administration of fruit juice in the LPS-induced inflammation model also resulted in the increased secretion of anti-inflammatory cytokines, such IL-10, and/or decreased levels of pro-inflammatory cytokines such as IL-1β, IL-6, and TNF-α in the murine peritoneal macrophage cultures [13].

Antioxidant activity might be another additional molecular mechanism of its anti-inflammatory effects. NF-κB activation is influenced by the cellular oxidative state, and antioxidants such as methyl chlorogenic acid (**13**) isolated from loquat leaf can inhibit the redox-sensitive NF-κB activation and downregulate NF-κB-dependent gene expression [19]. Treatment with loquat triterpene acids extracts significantly inhibited the methylene dianiline (MDA) level and the expression of heme oxygenase-1, and up-regulated the level of Superoxide dismutase (SOD) expression in cultured alveolar macrophages from CB rats [7]. Tormentic acid (**14**) from loquat suspension cells decreased paw edema in mice and increased the activities of catalase, SOD, and glutathione peroxidase in liver tissue [17].

### 2.2. Anti-Diabetic Activity

Diabetes mellitus is a metabolic disorder characterized by hyperglycemia resulting from either a defect in insulin secretion or action. As a traditional folk medicine component, *E. japonica* also exhibited great anti-diabetes potential. Recent research evidence has shown that loquat leaf or seed extracts are useful in prevention and control of both type-1 and type-2 diabetes [27,28,29,30,31,32,33].

**Figure 2:**
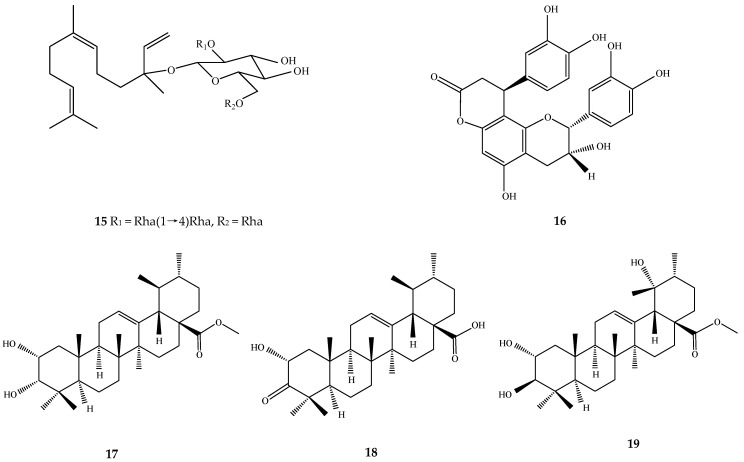
Structures of chemical compounds **15**–**19**. **15**: nerolidol-3-*O*-α-l-rhamnopyranosyl(1,4)-α-l-rhamnopyranosyl(1,2)-[α-l-rhamnopyranosyl(1,6)]-β-d-glucopyranoside; **16**: cinchonain Ib; **17**: 3-epicorosolic acid methyl ester; **18**: 2-α hydroxy-3-oxo urs-12-en-28-oic acid; **19**: tormentic acid methyl ester.

A 70% ethanol extract of *Folium Eriobotryae* (30 g/kg) showed significant hypoglycemic effect on alloxan-diabetic mice by lowering blood glucose levels [27]. By using the terpenes and flavonoids fraction of loquat leaf, their hypoglycemic potential on alloxan and/or streptozotocin (STZ)-induced diabetic mice was further investigated [27,29,30]. Results showed that the total triterpene acid fraction at 300 mg/kg day caused significant hypoglycemic and hypolipidemic effects on normal, alloxan and STZ-induced diabetic mice [29]. Total sesquiterpenes at 30 g/kg day showed similar hypoglycemic effects on alloxan-diabetic mice [27]. Further isolation of a triterpene acid—euscaphic acid (**6**)—and a sesquiterpene glycoside—nerolidol-3-*O*-α-l-rhamnopyranosyl(1,4)-α-l-rhamnopyranosyl(1,2)-[α-l-rhamnopyranosyl(1,6)]-β-d-glucopyranoside (**15**)—from *Folium Eriobotryae* showed that both compounds significantly lowered the plasma glucose levels in alloxan-diabetic mice, confirming that these are important active hypoglycemic constituents in loquat leaf (Figure 2) [34,35]. By using STZ-induced diabetic mice model, the flavonoid fraction containing quercetin-3-*O*-galactosyl-(1,6)-glucoside, quercetin-3-*O*-sophoroside, quercetin-3-*O*-rutinoside (rutin), kampferol-3-*O*-sophoroside, kampferol-3-*O*-rutinoside, querce-tin-3-*O*-galactoside (hyperoside), quercetin-3-*O*-glucoside (isoquercitrin), quercetin-3-*O*-rhamnoside, and kampferol-3-*O*-glucoside showed a hypoglycemic effect where a dose of 300 mg/kg significantly decreased the plasma glucose and serum insulin levels [30].

By using the high-fat (HF) diet-induced diabetic C57BL/6J mice model, the hypoglycemic effects of loquat extracts were also investigated [36,37]. Loquat leaf extract containing corosolic acid (**2**) and maslinic acid (**9**) significantly ameliorated the hyperglycemia, hyperleptinemia, and hyperinsulinemia in 45% HF diet C57BL/6J mice [36]. Cell suspension cultures of loquat contain a great number of pentacyclic terpenoids including tormentic acid (**14**), corosolic acid (**2**), ursolic acid (**1**), maslinic acid (**9**), and oleanolic acid (**8**) [37]. Addition of such cell suspension extract to the HF diet mice resulted in the prevention of the increase in the levels of blood glucose, insulin, leptin and homeostasis model assessment for insulin resistance index in the HF-diet mice [37]. By using the type 2 diabetic Otsuka Long–Evans Tokushima fatty (OLETF) rats and KK-A^y^ mice as experimental models, the hypoglycemic effects of loquat seeds were studied and results showed that OLETF rats fed a diet with 10% powdered loquat seed resulted in consistently reduced blood glucose concentration and serum insulin level compared to the control group [28]. In addition, ethanol extracts of loquat seed suppressed the increase in blood glucose for four months and improved the glucose tolerance in KK-A^y^ mice [28]. A new fermented tea product produced by co-fermentation of loquat leaf and summer-harvested green tea leaf (50 mg/kg) showed suppression of blood glucose level and a corresponding reduction in serum insulin secretion in maltose-loaded Sprague–Dawley (SD) rats [38]. Interestingly, such an effect was not observed when sucrose or glucose were administered to SD rats. Loquat leaf extracts led to a significant inhibition of the increase in serum glucose, total cholesterol and triglyceride levels induced in a hypercholesterolemic zebrafish model by feeding a high cholesterol diet [10].

In another study, a water extract of loquat leaf significantly increased the insulin secretion from INS-1 cells and decreased the insulin level for as long as 240 min post-administration in rats [39]. Cinchonain Ib (**16**) was found to enhance insulin secretion from INS-1 cells in the same study and may have insulinotropic effect for managing type 2 diabetes (Figure 2) [39].

Corosolic acid (**2**) isolated from loquat leaf promoted 3H-glucose uptake, inhibited the differentiation of preadipocytes into adipocytes, and downregulated the expression of peroxisome proliferator-activated receptor (PPAR)-γ and the CCAAT/enhancer binding protein-α in 3T3-L1 adipocytes [40]. Therefore, corosolic acid (**2**) might regulate carbohydrate metabolism without increasing adiposity [40]. Glucocorticoids are important regulators of metabolic processes including gluconeogenesis, and elevated glucocorticoids have been associated with hyperglycemia, insulin resistance and type 2 diabetes. Therefore, inhibition of the glucocorticoids-activating enzyme 11β-hydroxysteroid dehydrogenase 1 (11β-HSD1), which catalyzes the conversion of inactive 11-ketoglucocorticoids to active 11β-hydroxyglucocorticoids, is an important therapeutic target of antidiabetic medicines [41,42]. Among six traditional antidiabetic medicinal plants, loquat leaf extracts preferentially inhibited 11β-HSD1 [41]. The bioactivity-guided isolation of bioactive constituents resulted in the identification of corosolic acid (**2**), 3-epicorosolic acid methyl ester (**17**), 2-α hydroxy-3-oxo urs-12-en-28-oic acid (**18**), tormentic acid methyl ester (**19**), ursolic acid (**1**) as low micromolar inhibitors of 11β-HSD1 (Figure 2) [42].

### 2.3. Anti-Cancer Activity

As a traditional folk medicine component, loquat extracts have also displayed chemoprotective properties against various cancer cell lines. Modern science studies have demonstrated at the protein and gene level that loquat extracts can suppress cell carcinogenesis at different progression stages, such as cancer initiation, proliferation, and metastasis [43,44,45,46].

Both water and ethanol extracts of loquat leaf inhibited 7,12-dimethylbenz[α]anthracene (DMBA)-induced breast cancer in rats, and water extracts showed a higher inhibitory activity [46]. Both extracts inhibited the development of breast cancer by significantly suppressing the initiation and proliferation of tumor cells.

**Figure 3:**
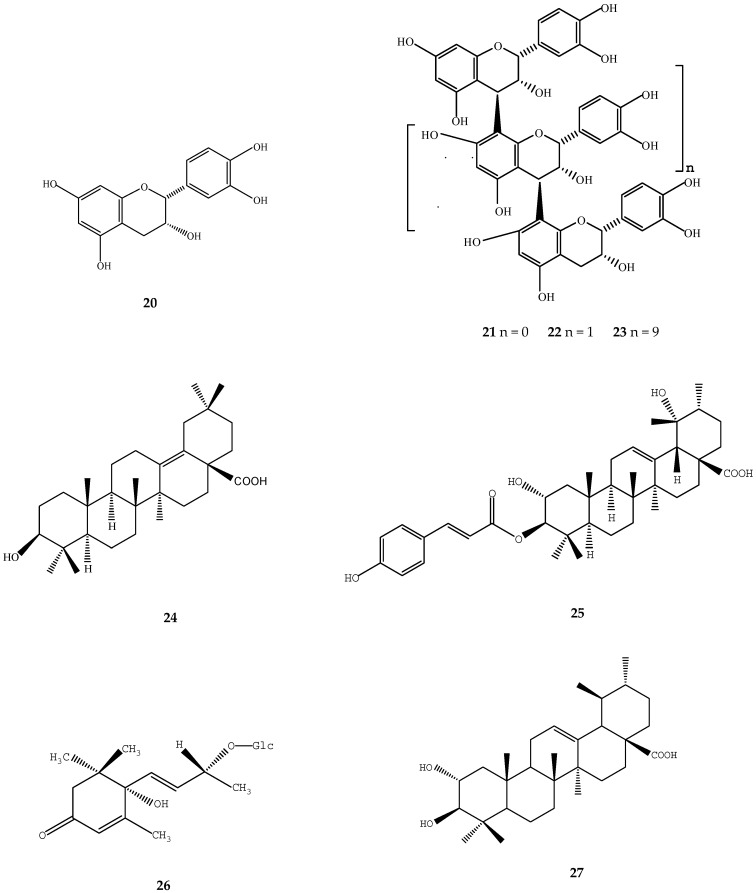
Structures of chemical compounds **20**–**27**. **20**: epicatechin; **21**: procyanidin B-2; **22**: procyanidin C-1; **23**: procyanidin oligomer; **24**: δ-oleanolic acid; **25**: 3-*O*-(*E*)-*p*-coumaroyl tormentic acid; **26**: roseoside; **27**: 2α-hydroxyursolic acid.

A large number of studies have demonstrated the cytotoxicity of loquat extract on different cancer cell lines. In an evaluation of 14 oriental medicinal herbs for antiproliferative activities, loquat leaf showed strong cytotoxicity in cell lines of estrogen receptor-negative breast cancer (MDA-MB-231), cervix epitheloid (HeLa) and lung (A549) carcinoma [47]. Ursolic acid (**1**) and oleanolic acid (**8**) isolated from loquat leaf significantly suppressed the proliferation of human lymphoid Molt 4B cells, which may result from the depletion of polyamines by inhibiting ornithine decarboxylase and *S*-adenosylmethionine decarboxylase activities [43]. Different procyanidin oligomers from loquat leaf showed selective cytotoxicity against human squamous cell (HSC-2) carcinoma and human salivary gland tumor cell [44]. Epicatechin (**20**), procyanidin B-2 (**21**), procyanidin C-1 (**22**), and procyanidin oligomer (**23**) showed increased cytotoxic activity against HSC-2 cells as molecular weight increased and such cytotoxic activity may be due to the prooxidant action of these polyphenols (Figure 3) [44]. Four triterpene acids, i.e., δ-oleanolic acid (**24**), ursolic acid (**1**), 3-*O*-(*E*)-*p*-coumaroyl tormentic acid (**25**), and betulinic acid (**12**) isolated from the methanol extracts of loquat leaf exhibited cytotoxicity against human HL60 cells (EC_50_ = 5.0–8.1 μM) and they also exhibited potent DNA topoisomerase I inhibition (IC_50_ = 20.3–36.5 μM) (Figure 3) [48]. Further study showed that 3-*O*-(*E*)-*p*-coumaroyl tormentic acid (**25**) induced apoptotic cell death in HL60 line mainly via the mitochondrial pathway and would be a promising compound for treatment of human leukemia [48].

By using a two-stage carcinogenesis assay on mouse skin, roseoside (**26**) isolated from loquat leaf was found to be the main compound that significantly delayed carcinogenesis induced by peroxynitrite as an initiator and TPA as a promoter (Figure 3) [49]. In another two-stage in vivo carcinogenesis test, euscaphic acid (**6**) showed significant antitumor promoting effects on mouse tumor induced by 7,12-DMBA as an initiator and TPA as a promoter [6].

Hydrophilic loquat extracts also showed in vivo anticancer activity in Meth-A-fibrosarcoma-bearing mice, operating through immunomodulatory activity, as indicated by factors such as interferon-gamma, interleukin-17, and TGF-β1 [45]. The possible constituents of such immunomodulatory activity require further investigation.

In addition, loquat leaf and seed extracts also showed significant anti-metastatic properties by inhibition of the migration and invasion of MDA-MB-231 human breast cancer cells and B16F10 melanoma cells, which was partially through the inhibition of matrix metalloproteinase-2 (MMP-2) and MMP-9 [22,24]. Ursolic acid (**1**) and 2α-hydroxyursolic acid (**27**) isolated from loquat extracts were indicated as key active compounds since both of them also significantly suppressed MMP-2 and MMP-9 activities (Figure 3) [24].

### 2.4. Antioxidant Activity

By using multiple antioxidant assay methods, diverse studies have demonstrated the strong antioxidant capacity of loquat extracts in vitro and in vivo. Both phenolic compounds and triterpene acids may contribute to such activity in different tissues of loquat.

The frequently reported antioxidant assay methods include Trolox equivalent antioxidant capacity (TEAC), 1,1-diphenyl-2-picrylhydrazyl radical (DPPH·) scavenging capacity, 2,2′-azinobis(3-ethylbenzothiazoline-6-sulphonic acid) (ABTS) assays, ferric reducing antioxidant power assay (FRAP), total antioxidant capacity, and extracts of loquat leaf, flower, fruit, and seed exhibited strong antioxidant capacity based on different assays [46,50,51,52,53,54]. Among 56 selected Chinese medicinal plants, loquat leaf showed higher antioxidant capacities than 54 other medicinal plants based on TEAC and FRAP assays [55]. The ABTS+ scavenging capacity of loquat flower was highly correlated with phenolics and flavonoids, and the correlation coefficients were 0.973 and 0.886, respectively [53]. Loquat fruit of different cultivars growing in different countries such as Turkey and China also showed significantly different antioxidant capacities, indicating the influence of both genetic background and growth environment on the accumulation of antioxidants [56,57].

High correlation between the antioxidant capacity and the total phenolic content were observed in loquat fruit of different cultivars grown in Turkey [58] or in China [5]. By dividing loquat fruit extract intro hydrophilic and lipophilic fractions, phenolic content and antioxidant activity of loquat fruit from 24 cultivars grown in China were investigated and the results showed that phenolic compounds are the major contributor to the hydrophilic antioxidant activity, while carotenoids were associated with the lipophilic antioxidant activity [52]. Loquat seed contained much higher content of polyphenolic compounds and showed stronger DPPH· scavenger activity than peel and pulp extracts [51].

**Figure 4:**
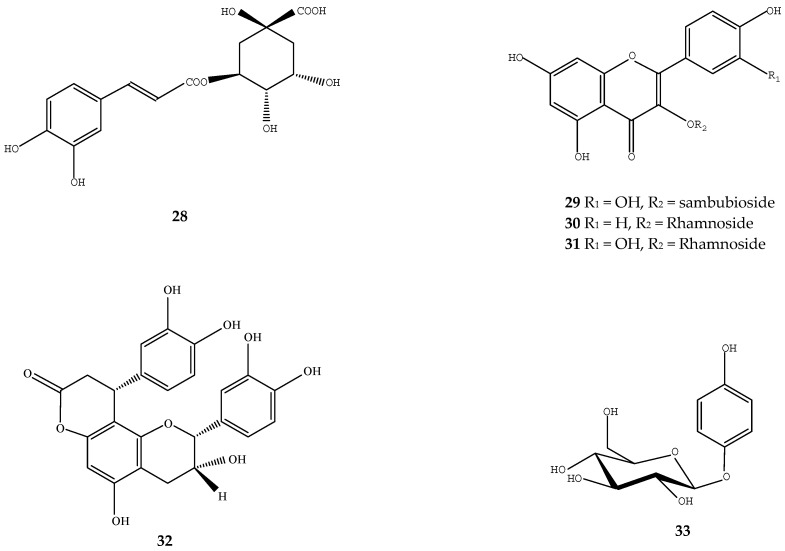
Structures of chemical compounds **28**–**33**. **28**: chlorogenic acid; **29**: quercetin-3-sambubioside; **30**: kaempferol-3-rhamnoside; **31**: quercetin-3-rhamnoside; **32**: cinchonain Ia; **33**: arbutin.

Chlorogenic acid (**28**) and quercetin-3-sambubioside (**29**), methyl chlorogenate (**13**), kaempferol-3-rhamnoside (**30**), quercetin-3-rhamnoside (**31**) isolated from loquat leaf extract all showed prominent inhibitory activity against free radical generation using the dichlorofluorescein method (Figure 4) [59]. The *n*-butanol, methanol and water fractions of loquat seed extract contained abundant polyphenols and showed high radical scavenging activity and inhibitory activity on lipid peroxidation, while the low-polar *n*-hexane and ethyl acetate fractions, which contained β-sitosterol, showed high lipid peroxidation inhibition activity [60]. In addition, ethanol extracts of loquat seeds were also effective in suppressing the oxidation of linoleic acid and the 2,20-azobis(4-methoxy-2,4-dimethylvaleronitrile)-induced low density lipoprotein oxidation [51]. In loquat leaf, cinchonain Ib (**16**), cinchonain Ia (**32**), epicatechin (**20**), quercetin-3-*O*-α-l-rhamnoside (**31**), and arbutin (**33**) have been identified as the important antioxidants exhibiting high antioxidant activity based on DPPH and FRAP assays (Figure 4) [61].

By using different cell models, different loquat fruit and leaf extracts showed protective effects against intracellular reactive oxygen species (ROS) [46,62,63]. Loquat fruit extract significantly inhibited the formation of ROS and NO in leukocytes and erythrocytes induced by the antibiotic chloramphenicol [63]. Ethanol extracts of loquat leaf showed hepatoprotective effects against ethanol-induced toxicity in HepG2 cells and a decrease in intracellular ROS formation, and increase in hepatic antioxidant activity, as well as increased cellular viability were observed [62]. Loquat leaf extract significantly increased antioxidant enzyme activities of SOD, catalase, glutathione-*S*-transferase, glutathione peroxidase, glutathione reductase, reduced glutathione in HepG2 cells [62]. In another cell model using β-amyloid-induced oxidative stress in neuronal PC12 cells, treatment of loquat leaf efficiently suppressed the formation of intracellular ROS formation by Aβ_1-42_ peptide and inhibited neuronal cell death [46]. Ursolic acid (**1**) in loquat leaf was reported to increase catalase activities in mouse liver [64].

By using animal models, loquat seed extracts were found to significantly reduce oxidative stress in adriamycin-induced nephropathy in rats by increasing the reduced glutathione levels in renal tissue and lowering the lipid peroxide levels in plasma and renal tissue [65]. In addition, loquat seed extract enhanced antioxidant enzyme activity and reduced lipid peroxidation in liver tissue of rats with non-alcoholic steatohepatitis [66].

### 2.5. Other Bioactivities

Loquat extracts have shown other bioactivities such as improvement of liver function [66,67], lung [68], renal [65] and neuronal cells [46], and also loquat extracts showed anti-obesity and hypolipidemic activity [36,37,69], anti-thrombotic potential [20], antiaging effects [70], anti-allergic [22], and antinociceptive activities [24], etc.

Both the 70% ethanol and methanol extracts of loquat seed showed significant inhibition of the development of liver fibrosis in the dimethylnitrosamine-induced hepatopathic rats and the loquat extracts significantly decreased the level of l-asparate aminotransferase (AST), l-alanine aminotransferase (ALT), and hydroxyproline levels and increased the retinoid levels in hepatopathic rats [67]. The unsaturated fatty acids linolenic and linoleic acids and the sterol β-sitosterol within the extracts may contribute to such hepatoprotective functions [67]. Long-term heavy consumption of alcohol may result in the development of alcohol related liver disease, where the induction of cytochrome P-450 2E1 (CYP2E1) by ethanol could result in a state of oxidative stress. Loquat leaf extracts showed hepatoprotective effects in HepG2 cells overexpressing CYP2E1 by improving the hepatic antioxidant activity and decreasing the formation of intracellular ROS [62]. As a result, loquat leaf extract increased HepG2 cell viability in a concentration-dependent manner and showed protective activity against ethanol-induced toxicity in HepG2 cells [62]. In addition, loquat seed extracts also showed protective activity against non-alcoholic steatohepatitis. The 70% ethanol extract of loquat seed significantly inhibited the increases in ALT and AST levels and the formation of fatty droplets in the liver in rats [66]. Such effects of inhibition of fatty liver and fibrosis may result from the elevated antioxidant enzyme activity that may alleviate oxidative stress in rats with non-alcoholic steatohepatitis [66].

**Figure 5:**
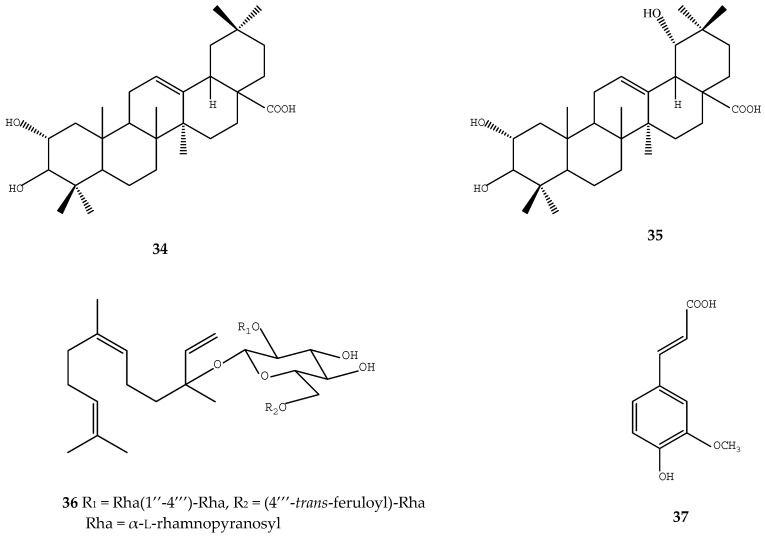
Structures of chemical compounds **34**–**37**. **34**: *α*-hydroxyoleanolic acid; **35**: arjunic acid; **36**: 3-*O*-α-l-rhamnopyranosyl-(l→4)-α-l-rhamnopyranosyl-(1→2)-[α-l-(4-*trans*-feruloyl)-rhamnopyranosyl-(1→6)]-β-d-glucopyranosyl nerolidol; **37**: ferulic acid.

By using a rat model of bleomycin-induced pulmonary fibrosis, triterpene acids of loquat leaf showed antifibrosis effects by ameliorating the lung structure and alleviating fibrosis in the rat, where reduced expression of TNF-*α* and TGF-β1 both at the protein and mRNA levels were observed in the alveolar macrophage of pulmonary fibrosis rats [68]. The triterpene acids present in such loquat extracts included oleanolic acid (**8**), α-hydroxyoleanolic acid (**34**), arjunic acid (**35**), euscaphic acid (**6**), and ursolic acid (**1**), etc. (Figure 5) [68].

Anticancer agents such as adriamycin cause adverse effects such as renal and liver disorders. Studies showed that loquat seed extracts could improve renal function disorder caused by adriamycin in rats and such anti-nephropathy activity may result from the alleviated oxidative stress caused by the antioxidant properties of loquat extracts [65]. Treatment with loquat extract significantly increased the level of reduced glutathione in renal tissue and lowered the level of lipid peroxide in plasma and renal tissue of adriamycin-induced nephropathic rats [65].

Oxidative stress is believed to be involved in the pathogenesis of neurodegenerative disorders. Loquat extract showed neuroprotective effects against Aβ peptide-induced oxidative stress and inhibited Aβ_1–42_-mediated neuronal cell death in vitro, restored the alternation behavior and reversed the Aβ_1–42_-induced memory impairment in mice [46]. The loquat extract substantially inhibited lipid peroxidation and restored SOD activity and cognitive deficit induced by Aβ peptide [46].

The pentacyclic terpenoids extracts of cell suspension culture of loquat showed anti-hyperlipidemic effects in HF diet mice since it reduced body weight gain, weights of white adipose tissue (WAT) (including epididymal, perirenal, mesenteric WAT and visceral fat), size of adipocytes in the visceral depots and hepatic triacylglycerol content [36,37]. Such anti-hyperlipidemic effects were correlated with increased protein phosphorylation of AMPK-α (Thr172) both in liver and adipose tissue, increased the adipose PPARγ and hepatic PPARα mRNA levels, and decreased gene expressions of fatty acid synthesis, including acyl-coenzyme A: diacylglycerol acyltransferase 2 [36,37]. Fermented tea product from a combination of loquat leaf and *Camellia sinensis* green tea leaf exhibited hypotriacylglycerolemic and antiobesity properties through suppression of fatty acid synthesis in liver and postprandial hypertriacylglycerolemia by inhibition of pancreatic lipase [69]. Loquat leaf showed anti-atherosclerotic activity in cellular assays and in a hypercholesterolemic zebrafish model [10].

The membrane glycoprotein thromboplastin is a tissue factor that accelerates blood clotting. Based on a bioassay-directed chromatographic separation technique, a sesquiterpene glycoside, namely 3-*O*-α-l-rhamnopyranosyl-(l,4)-α-l-rhamnopyranosyl-(1,2)-[α-l-(4-*trans*-feruloyl)-rhamnopyranosyl-(1,6)]-β-d-glucopyranosyl nerolidol (**36**) and ferulic acid (**37**) were identified from the loquat leaf as active compounds that can inhibit 50% of the tissue factor at concentration of 2 and 369 µmol/L, respectively, and thus loquat extracts may have great anti-thrombotic potential (Figure 5) [20].

Loquat seed extract also showed antiaging activity by ameliorating the cellular aging in cultured rat fibroblasts [70]. Treatment of loquat extract for seven days rendered bradykinin (BK)-induced Ca^2+^ dynamics in senescence cells similar to those in young cells, and retarded and/or protected against cellular aging [70]. The antiosteoporosis effect of loquat leaf was also reported in an ovariectomized mice model [71]. In another study, loquat leaf extract at 50, 125, 250, and 500 μg/mL showed inhibition of the differentiation of osteoclasts and ursolic acid (**1**) was isolated as the key compound that inhibited osteoclast, based on a bioactivity-guided fractionation [71].

By using the mast cell-mediated anaphylactic reaction as an experimental model, loquat leaf extract was found to inhibit systemic anaphylactic reactions and histamine release from mast cells in mice [22]. It inhibited the production of TNF-α in phorbol 12-myristate 13-acetate and A23187-stimulated human mast cells and was therefore considered as a good candidate as an anti-allergic resource [22]. The *n*-butyl alcoholic fraction of loquat leaf also demonstrated excellent antinociceptive activity in a dose-dependent manner in experimental pain models and it may act as a weak opioid agonist [24].

## 3. Data Collection

Our research included a thorough investigation of published literature in order to collect a significant amount of information related to loquat and its biological activities. The main keywords we used were loquat, fruit, seed, leaf, health, biological activity, inflammatory, diabetes, cancer, antioxidant, phenolics, terpenoids, chemical structure, etc. The databases searched included Pubmed and Web of Science.

## 4. Conclusions

A range of bioactivities has been reported for different loquat extracts, a number of bioactive compounds have been identified, and active research is continuing. Studies investigating in vivo metabolism and bioavailability, synergies and competitive effects, and potential toxicity of loquat extracts in animal or cell models are receiving more attention. Since many important compounds such as ursolic acid (**1**), chlorogenic acid (**28**), quercetin glycosides (**29**,**31**), and its derivatives have been well studied for their bioactivities as pure chemical compounds, irrespective of the source, eating raw loquat fruit or its processed food products may have similar health-benefiting effects. Furthermore, new applications for different loquat organs as ingredients for functional foods or as a source of therapeutics are anticipated. Extensive studies should be carried out on structure–activity relationships for different bioactive compounds. In addition, breeding and genetic studies of loquat to increase the accumulation of pharmaceutically active compounds for human health may provide a new focus for loquat research and industry.

## Abbreviations

ABTS	2,2′-Azinobis(3-ethylbenzothiazoline-6-sulphonic acid)
ALT	l-Alanine aminotransferase
AST	l-Asparate aminotransferase
CB	Chronic bronchitis
COX-2	Cyclooxygenase-2
CYP2E1	Cytochrome P-450 2E1
DMBA	Dimethylbenz[α]anthracene
DPPH	1,1-Diphenyl-2-picrylhydrazyl
ERK	Extracellular signal-regulated kinase
FRAP	Ferric reducing antioxidant power
HF	High-fat
HSC	Human squamous cell
11β-HSD1	11β-Hydroxysteroid dehydrogenase 1
IL-10	Interleukin-10
IL-1*β*	Interleukin-1*β*
IL-6	Interleukin-6
IL-8	Interleukin-8
iNOS	Inducible nitric synthase
LPS	Lipopolysaccharide
MAPK	Mitogen-activated protein kinase
MDA	Methylene dianiline
MMP	Matrix metalloproteinase
NF-κB	Nuclear factor-κB
NO	Nitric oxide
OLETF	Otsuka Long–Evans Tokushima fatty
PPAR	Peroxisome proliferator-activated receptor
ROS	Reactive oxygen species
SD	Sprague–Dawley
SOD	Superoxide dismutase
STZ	Streptozotocin
TEAC	Trolox equivalent antioxidant capacity
TGF-*β*1	Transforming growth factor-beta 1
TNF-*α*	Tumor necrosis factor-*α*
TPA	12-*O*-tetradecanoylphorbol-13-acetate
WAT	White adipose tissue
